# Electrical monitoring of photoisomerization of block copolymers intercalated into graphene sheets

**DOI:** 10.1038/s41467-020-15132-z

**Published:** 2020-03-12

**Authors:** Semin Kim, Thanh-Hai Le, Yunseok Choi, Haney Lee, Eunseo Heo, Unhan Lee, Saerona Kim, Subin Chae, Yoong Ahm Kim, Hyeonseok Yoon

**Affiliations:** 1 Department of Polymer Engineering, Graduate School, Chonnam National University, 77 Yongbong-ro, Buk-gu, Gwangju 61186, South Korea; 2 Alan G. MacDiarmid Energy Research Institute & School of Polymer Science and Engineering, Chonnam National University, 77 Yongbong-ro, Buk-gu, Gwangju 61186, South Korea

**Keywords:** Polymers, Polymers, Molecular self-assembly

## Abstract

Insulating polymers have received little attention in electronic applications. Here, we synthesize a photoresponsive, amphiphilic block copolymer (PEO-*b*-PVBO) and further control the chain growth of the block segment (PVBO) to obtain different degrees of polymerization (DPs). The benzylidene oxazolone moiety in PEO-*b*-PVBO facilitated chain-conformational changes due to photoisomerization under visible/ultraviolet (UV) light illumination. Intercalation of the photoresponsive but electrically insulating PEO-*b*-PVBO into graphene sheets enabled electrical monitoring of the conformational change of the block copolymer at the molecular level. The current change at the microampere level was proportional to the DP of PVBO, demonstrating that the PEO-*b*-PVBO-intercalated graphene nanohybrid (PGNH) can be used in UV sensors. Additionally, discrete signals at the nanoampere level were separated from the first derivative of the time-dependent current using the fast Fourier transform (FFT). Analysis of the harmonic frequencies using the FFT revealed that the PGNH afforded sawtooth-type current flow mediated by Coulomb blockade oscillation.

## Abstract (web summary)

Block copolymers are electrically insulating and therefore characterization with electrical or electrochemical methods is not possible. Here, the authors demonstrate electrical monitoring of the photoisomerization transition in a benzylidene oxazolone block co-polymer intercalated into graphene sheets.

## Introduction

Synthesis of block copolymers with controlled characteristics has been extensively studied in recent decades. Several important polymerization strategies have been developed, such as conventional radical polymerization, ionic polymerization, and controlled radical polymerization. In particular, atom transfer radical polymerization (ATRP), a type of controlled radical polymerization, has been widely used to create block copolymers with controlled components and degrees of polymerization (DPs)^1–3^. Advances in polymerization techniques have made it possible to produce functional polymers for a variety of applications. For example, many different types of stimuli-responsive polymers have been synthesized, which are sensitive to changes in environmental parameters such as pH, temperature, humidity, pressure, and light. Specific functional groups targeting specific properties have been site-selectively incorporated into these polymers. Typically, photoresponsive groups, such as azobenzene, spiropyran, dithienylethene, oxazolone, and their derivatives, have been employed in light-responsive polymers. In particular, the oxazolone group, which acts as a chromophore in green fluorescent protein, undergoes *Z*‒*E* isomerization upon light irradiation^4–9^.

Coulomb-blockade phenomena in nanostructures have also attracted much attention in condensed matter physics. Prototypical examples include a quantum dot separated from source/drain leads by tunnel junctions in a transistor configuration, where electrons or holes can be injected individually into the quantum dot by varying the gate voltage^10–15^. The unique transport behavior in the Coulomb-blockade regime may lead to single-spin quantum bits or coherent many-body interactions between the confined spin and the Fermi reservoir, depending on the tunnel coupling strength. Likewise, Coulomb-blockade phenomena have been investigated mainly for small-area tunnel junctions in sophisticated microelectrode configurations^10,16–19^.

Because most block copolymers are electrically insulating, electronic, electrical, or electrochemical characterization has not been possible. Consequently, block copolymers may have many unexplored but intriguing electronic/electrical characteristics. In this work, we synthesize amphiphilic block copolymers including the benzylidene oxazolone (BO) moiety in the repeat unit and are able to electrically monitor the photoisomerization transition of the insulating block copolymer by intercalating it into graphene sheets^20–22^. The BO-containing block copolymer/graphene nanohybrids can be directly used in amperometric ultraviolet (UV) sensors^23–27^. Furthermore, we observe block-copolymer-mediated Coulomb-blockade phenomena in the stacked graphene sheets. We also demonstrate the dependence of the Coulomb-blockade oscillations on the DP of the block copolymer. These results show great promise for facilitating wider applications of Coulomb-blockade phenomena.

## Results

### Synthesis of block copolymers with controlled DPs

**Fig. 1:**
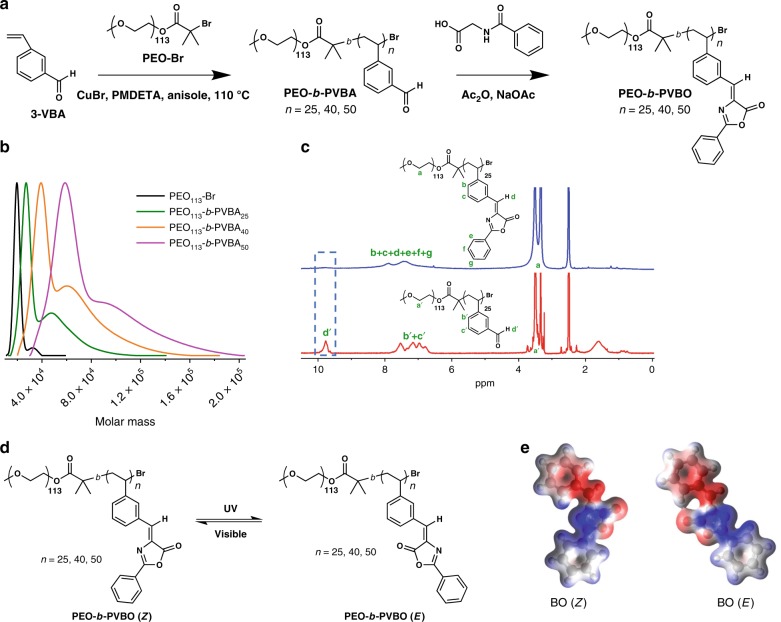
Synthesis and characterization of PEO-*b*-PVBOs. **a** Synthesis of block copolymers with pendant BOs. **b** Overlaid GPC traces of PEO-Br and a series of PEO-*b*-PVBA block copolymers. **c** ^1^H NMR spectra of PEO_113_-*b*-PVBA_25_ (red) and PEO_113_-*b*-PVBO_25_ (blue). **d** Light-induced isomerization between PEO-*b*-PVBO (*Z*) and PEO-*b*-PVBO (*E*) under alternating UV and visible light irradiation. **e** Energy density mapping of the isomers calculated at the B3LYP/6-311+G(d,p) level of theory.

A photoresponsive, amphiphilic block copolymer was designed and synthesized, as shown in Fig. 1. The synthetic strategy is illustrated in Fig. 1a. The light-responsive 3-vinylbenzaldehyde (3-VBA) block was extended from a PEO-Br macroinitiator via ATRP. Anisole was used as a solvent, and the copper(I) bromide/*N*,*N*,*N*′,*N*′′,*N*′′-pentamethyldiethylenetriamine (PMDETA) catalyst system was employed for block copolymerization. The initial ratio of 3-VBA to the PEO-Br macroinitiator was 100:1. Samples were removed at regular intervals (2 h) during polymerization to obtain PVBA blocks with different lengths and to monitor the conversion and molecular weight evolution. The resulting poly[(ethylene oxide)-*b*-poly[(3-vinylbenzaldehyde)] (PEO-*b*-PVBA) copolymers were analyzed by gel permeation chromatography (GPC) and ^1^H nuclear magnetic resonance (NMR) spectroscopy using dimethyl sulfoxide (DMSO)-d_6_ as the solvent. The GPC traces shifted continuously toward higher molecular weight throughout the polymerization, indicating well-controlled block copolymerization (Fig. 1b). The DPs of a series of PEO-*b*-PVBA copolymers were also obtained experimentally from the ^1^H NMR spectra (DP_n, NMR_) by calculating the integration area of the PEO repeat unit signals at 3.5 ppm and the aldehyde signals of the PVBA block at 9.9 ppm. The calculated DPs of the PVBA blocks were 25, 40, and 50. Then, the aldehyde group of PEO-*b*-PVBA was converted to BO by reacting with hippuric acid in the presence of sodium acetate, yielding the poly[(ethylene oxide)-*b*-poly[(3-vinylbenzylideneoxazolone)] (PEO-*b*-PVBO) block copolymer. The ^1^H NMR spectra provided quantitative evidence of post-polymerization modification, as the aldehyde peaks of VBA at 9.9 ppm disappeared completely (Fig. 1c).

The chromophore BO has characteristic photoswitching behavior based on geometrical isomerism under UV and visible light irradiation. The introduction of the BO moiety into a block segment enabled a unique type of chain-conformational change in the PEO*-b-*PVBO by geometrical isomerization under irradiation at different wavelengths. The photoluminescence decay behavior of the PEO*-b-*PVBO also depended on the DP of the PVBO block. (Supplementary Fig. 1). Figure 1d shows the *Z*‒*E* isomeric transition of PEO*-b-*PVBO induced by UV and visible light. The different geometrical configurations of the *E* and *Z* isomers result in different chain-conformational structures of the block copolymers. In addition, the ester moiety of the *E* isomer is close to the adjacent benzene ring, whereas the ester moiety of the *Z* isomer is relatively free from any steric hindrance. The oxygen atoms in the ester moiety of the BO have lone-pair electrons. Therefore, light-induced isomerization can further alter significant characteristics of the PEO-*b*-PVBO, such as the chemical reactivity and electron density distribution. Density functional theory was used to calculate the electronic structures of the isomers (Fig. 1e). The electron density distributions of the two isomers are clearly different. Remarkably, the red region representing the C=O group of the *E* isomer overlapped the blue region representing the protons of the adjacent benzene ring, indicating that the C=O group was bound to the proton of the benzene via physical bonding. By contrast, no intermolecular interaction appeared in the interior of the *Z* isomer. These calculated data confirmed that photoisomerization triggered by the different light sources can induce significant changes in the chemical and electronic properties of PEO-*b*-PVBO, as well as its geometrical structure.

### Intercalation of PEO-*b*-PVBO into graphene sheets

**Fig. 2:**
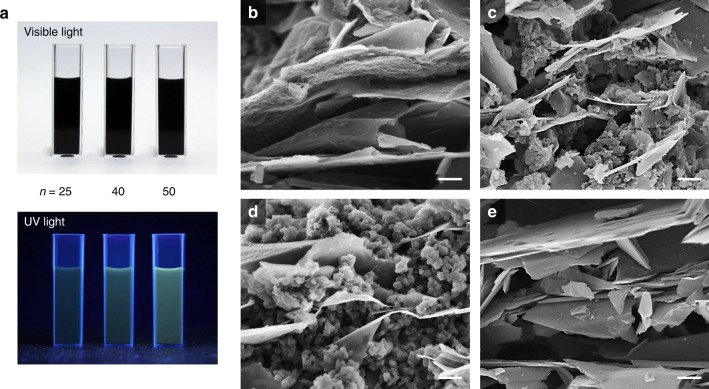
Characterization of PGNHs. **a** Photographs showing the color changes of the P_*n*_GNH dispersions (*n* = 25, 40, and 50) under visible and UV light illumination. **b**–**e** SEM images of P_*n*_GNHs with **b** *n* = 25, **c** *n* = 40, and **d** *n* = 50, and **e** control (graphene sheets alone). The scale bar is 200 nm.

By using ATRP, it was possible not only to synthesize well-defined PEO*-b-*PVBO but also to control the DP of the PVBO block. Therefore, PEO*-b-*PVBO itself is useful for practical applications such as photodetectors and photoswitches. However, to fully explore its properties, the block copolymer was hybridized with another nanomaterial, namely, graphene. PEO*-b-*PVBO was successfully intercalated into the confined space between graphene sheets by physical exfoliation of graphite in an aqueous medium. The PEO*-b-*PVBO-intercalated graphene nanohybrids (PGNHs) are labeled P_*n*_GNH and PEO_*m*_*-b-*PVBO_*n*_, where *m* and *n* denote the DPs of the PEO and PVBO blocks, respectively. As mentioned earlier, the DP of the PEO block was fixed at a constant value (113), whereas the PVBO blocks had various DPs (25, 40, and 50). Note that physical exfoliation of graphite preserves the main properties of the resulting graphenes. Further, most block copolymers, including PEO-*b*-PVBO, are electrically insulating; therefore, they have not been used directly in electronic/electrical applications to date. However, intercalating PEO-*b*-PVBO into graphene sheets during physical exfoliation offered a unique opportunity for electrical monitoring of the behavior of the block copolymer at the molecular level. The PGNHs showed good colloidal stability in aqueous solution, which was in contrast to the poor colloidal stability of the physically exfoliated graphene sheets used as a control. Graphene itself is inherently hydrophobic. Thus, the stable aqueous-phase dispersion of the PGNHs indicates that the amphiphilic PEO*-b-*PVBO was adsorbed on graphene sheets, improving the colloidal stability. Figure 2a shows the aqueous colloidal dispersions of PGNHs under visible and UV irradiation. Under visible light illumination, the aqueous PGNH dispersions appeared black. By contrast, under UV illumination, the dispersions became dark yellow, and the intensity of the color depended on the DP of the PVBO block. The yellow color appeared more intense with increasing DP of the PVBO block in the PEO-*b*-PVBO, indicating that the PVBO blocks intercalated between the graphene sheets functioned as a chromophore. Figures 2b–e displays scanning electron microscopy (SEM) images of PGNHs and the control (graphene sheets alone). In all the PGNHs, PEO*-b-*PVBO was well intercalated into the graphene sheets. In addition, it is noteworthy that the morphology of the intercalated PEO*-b-*PVBO depended on the DP of the PVBO block. The PEO block is hydrophilic, and the PVBO block is hydrophobic. Therefore, the hydrophilic–lipophilic balance decreases with increasing DP of the PVBO block. P_25_GNH was relatively hydrophilic, as it was found to be adsorbed on graphene in the form of somewhat amorphous, rough films. By contrast, particulate PEO-*b*-PVBO aggregates were observed between the graphene sheets for PVBO blocks with higher DPs. In particular, almost spherical nanoparticles (~50 nm in diameter) were found in P_50_GNH, indicating that the PEO_113_-*b*-PVBO_50_ block copolymer was sufficiently hydrophobic for self-assembly into spheres due to high interfacial tension in the aqueous phase. In other words, the morphology of the PEO-*b*-PVBO block copolymer intercalated between the graphene sheets in aqueous solution evolved from a film to particulates with increasing DP of the PVBO block.

### Photoisomerization behavior

**Fig. 3:**
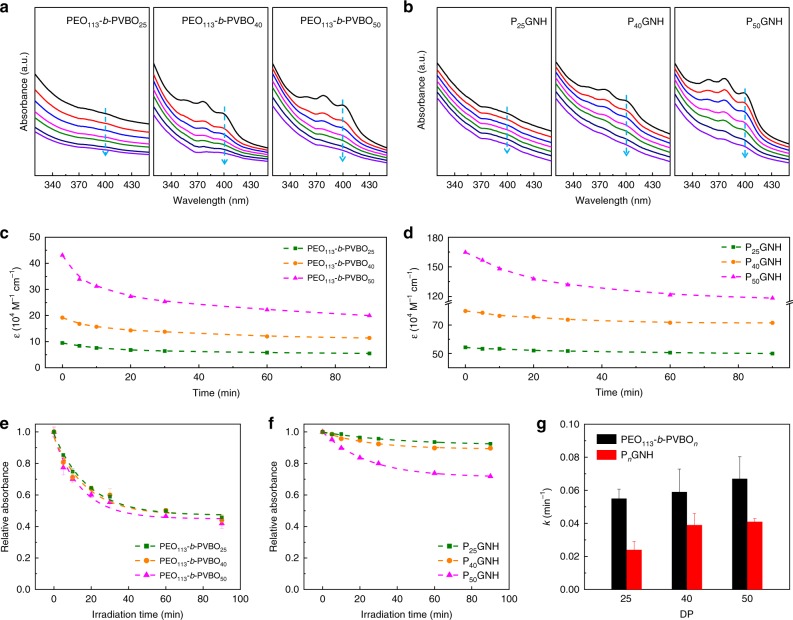
Photoisomerization behavior under UV/visible illumination. UV–visible absorption spectra of aqueous dispersions of (**a**) PEO_113_-*b*-PVBO_*n*_ and (**b**) P_*n*_GNH under UV irradiation for 90 min (recorded at 0, 5, 10, 20, 30, 60, and 90 min). Molar extinction coefficients at 400 nm as a function of irradiation time for (**c**) PEO_113_-*b*-PVBO_*n*_ and (**d**) P_*n*_GNH. Relative absorbance [*A*(*t*)/*A*_0_] of the major peak at 400 nm as a function of irradiation time, where the absorbance *A* was divided by the initial value *A*_0_, for (**e**) PEO_113_-*b*-PVBO_*n*_ and (**f**) P_*n*_GNH. **g** Calculated rate constants (with s.d. error bars) for different DPs.

UV–visible spectroscopy was used to examine the photoisomerization behavior of the PEO-*b*-PVBO alone and PGNHs in aqueous solution (Fig. 3a, b). The absorption spectra of the block copolymers and the block-copolymer-intercalated graphene sheets first reached equilibrium states under visible light illumination, and then their changes in absorption under UV illumination were monitored for 90 min. Under visible light illumination, PEO_113_*-b-*PVBO_25_ showed only indistinct, weak absorption at 370‒420 nm. By contrast, PEO_113_*-b-*PVBO_40_ and PEO_113_*-b-*PVBO_50_ revealed three major absorption peaks at ~356, 376, and 400 nm (similar absorption behavior was observed from PVBO homopolymer, as shown in Supplementary Fig. 2), where the peaks of PEO_113_*-b-*PVBO_50_ were more intense than those of PEO_113_*-b-*PVBO_40_. The dependence of the absorption intensity on the DP of the PVBO block under visible illumination confirms that the BO moiety of the PVBO block works well as a chromophore in the block copolymer. Then, under UV irradiation, the characteristic absorption peaks gradually decreased, indicating that the chromophoric behavior of the BO moiety of the PVBO block was screened via a conformational change in the chain during photoisomerization. The same trend was also observed in the absorption spectra of the PGNHs. Compared to PEO-*b*-PVBOs, as plotted in Fig. 3c, d, PGNHs had higher molar extinction coefficients. The graphene sheets are thought to interfere with the absorption of the block copolymer (Supplementary Fig. 3). Consequently, the level of the changes became slightly lowered when compared to the block copolymer alone. The dependence of the photoisomerization rate on the DP of the PVBO block was examined by kinetically quantifying the changes in absorbance. As a typical absorption peak (see Supplementary Fig. 4), the absorbance at 400 nm is plotted as a function of time in Fig. 3e, f. The rate of *Z*–*E* photoisomerization can be described by a first-order reaction,

1 $$\left[ {Z\left( t \right)} \right]/\left[ Z \right]_0 = e^{ - kt}$$

where *Z* and *k* denote the *Z* isomer of PEO-*b*-PVBO and the rate constant, respectively. The absorbance is the sum of the absorbance of the individual components (the PVBO block, PEO block, and graphene). Considering that the absorbance of the PEO block and graphene would remain constant during photoisomerization, the absorbance *A* can be correlated with the concentration of the *Z* isomer using the following equation (see details in Supplementary Information):

2 $$A\left( t \right)/A_0 = \beta + \alpha e^{ - kt}$$

where *α* and *β* are constants, and thus the rate constant is calculated by fitting the relative absorbance curves to Eq. (2). As shown in Fig. 3g, the rate constant for photoisomerization increased with increasing DP of the PVBO block both with and without graphene. The rate constants of the PGNHs were 34–56% lower than that of PEO-*b*-PVBO alone. Note, however, that the ability of PEO-*b*-PVBO to exhibit photoisomerization was well maintained even after its intercalation into the confined space between graphene sheets.

### Photoswitching characteristics

**Fig. 4:**
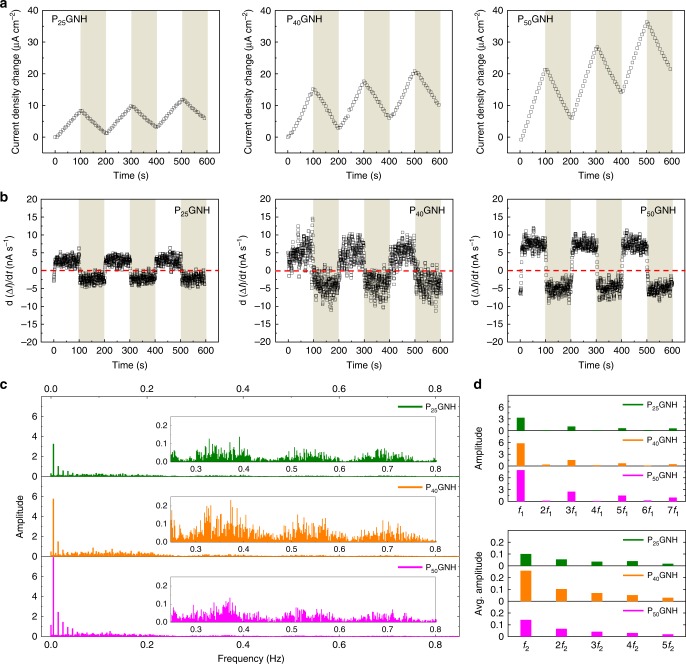
Electrical responses of PGNH electrodes under periodic exposure to visible and UV light. **a** Plots of current density change (Δ*I*) versus time. **b** Plots of d(Δ*I*)/d*t* versus time. **a, b** Visible and UV light are indicated by white and gray regions, respectively. **c** FFT analysis data from the plots in (**b**) (inset: magnified view of data at 0.25‒0.80 Hz). **d** Amplitudes and average amplitudes at the fundamental frequency peak *f*_1_ (5 mHz) and in the fundamental frequency band *f*_2_ (174 ± 25 mHz), respectively, and their harmonics obtained from (**c**).

Intercalation of the PEO-*b*-PVBO into graphene sheets offered a unique opportunity to electrically monitor the photoisomerization. Next, a circular pattern of block-copolymer-intercalated graphene sheets was deposited between two gold electrodes on a glass substrate using a polydimethylsiloxane (PDMS) mask. Importantly, a series-connection-like configuration of small PEO-*b*-PVBO/graphene pieces was formed over the gap between the gold electrodes to amplify changes in the electrical current flowing through the electrodes^24^. First, the photoisomerization behavior was electrically monitored by measuring current as a function of time at a constant applied voltage. As shown in Fig. 4a, the PGNHs showed photoswitchability upon periodic exposure to visible/UV light (see the response for only graphene in Supplementary Fig. 5). The chain-conformational change in the intercalated PEO-*b*-PVBO due to photoisomerization would not only alter the main properties of the block copolymer but also cause physical perturbation in the confined space between graphene sheets. The current change (Δ*I* = *I* − *I*_0_, where *I*_0_ and *I* are the initial current and real-time current, respectively) was positive under visible light illumination but negative under UV light illumination. In other words, UV illumination led to an increase in the resistance of the PGNH electrode, indicating that the chain-conformational change of PEO-*b*-PVBO by *Z*‒*E* photoisomerization had a negative effect on current flow through the electrode. The rate of current change depended on the DP of the PVBO block. The first derivative of the current change as a function of time, d(Δ*I*)/d*t*, clearly indicated that the current increased under visible illumination, whereas it decreased under UV illumination (Fig. 4b). The rate of current change was also proportional to the DP of the PVBO block. Hence, it is important to note that the electrical signal was quantitatively correlated with the chain-conformational change of the block copolymer, which occurs at the molecular level. In addition, this type of photoswitching behavior enables direct application of the PGNH electrode in UV sensors. Note that the PGNH electrode is very easy and cost-effective to fabricate, whereas most UV sensors require delicate materials and sophisticated device systems.

### Coulomb-blockade oscillation

More interestingly, Coulomb-blockade oscillation was observed in the current flowing through the PGNH electrode. The current oscillated at the level of a few nanoamperes under both visible and UV illumination, as seen in Fig. 4b, where the oscillation behavior appeared to depend on the DP of the PVBO block. Photoisomerization of the block copolymer chains confined between the graphene sheets induced a large periodic square-type change in the current. In addition, under visible or UV illumination, continuous current oscillation was observed at smaller scales. PEO-*b*-PVBO is a resistor, and when it is embedded in conductive graphene sheets, only tunneling is allowed between geometrically separated graphene sheets. The tunneling can be affected by the photoisomerization of the PEO-*b*-PVBO accompanying its conformational change at the confined, ultrasmall space of the inter-graphene sheets. More specifically, physical perturbation by the conformational change of PEO-*b*-PVBO at the confined space can affect the tunneling. In addition, as given in Fig. 1e, the isomers had different (localized) electron density distributions, which can also alter the tunneling through variation in Coulombic interaction. Therefore, the variation in chemical/electronic characteristics of the tunnel junction by the physical rearrangement of the blocks would affect the tunneling. In addition, a series-connection-like configuration of intercalated PEO-*b*-PVBO resistors was formed when a voltage was applied perpendicular to the stacked graphene sheets. Importantly, the series combination of the resistors along the *z*-axis of stacked graphene sheets would contribute to electrically amplifying the responses from the intercalated block copolymer[24]. Fast Fourier transform (FFT) analysis was applied to the first derivative of the time-dependent current to investigate the periodicity and amplitude of the Coulomb-blockade oscillation, as shown in Fig. 4c. Two types of oscillation were detected from the frequency domains (see Fig. 4d and Supplementary Fig. 6). First, a high-amplitude frequency peak at 5 mHz (*f*_1_) was found, followed by odd harmonic frequency peaks (e.g., 3*f*_1_, 5*f*_1_, and 7*f*_1_). This type of series of frequency peaks can be attributed to the large square-type oscillation in the time domain due to photoisomerization. Next, a frequency band covering a range of ±25 mHz centered at 174 mHz (*f*_2_) was found in the low-frequency domain, which repeats periodically at 50 mHz intervals. The block copolymers were randomly intercalated between the graphene sheets, so the frequency bands would cover a specific range of frequencies. Frequency bands that are positive integer multiples of the original frequency band centered at 174 mHz were observed, indicating that sawtooth-type Coulomb-blockade oscillation occurred. The amplitude of the Coulomb-blockade oscillation also varied with the DP of the PVBO block, indicating that PVBO significantly contributed to the Coulomb-blockade transport.

## Discussion

We have successfully synthesized the photoresponsive, amphiphilic block copolymer PEO_113_-*b*-PVBO_*n*_ via ATRP, where the DP of the PVBO block was controlled to one of three values (*n* = 25, 40, or 50). The PVBO block facilitated *Z*–*E* photoisomerization under visible/UV light irradiation. The PEO-*b*-PVBOs were physically intercalated into graphene sheets to minimize degradation of the properties of graphene, providing an opportunity to directly observe the chain-conformational change of the block copolymer by monitoring the electrical signal. Furthermore, the current change of the PGNH under alternating visible/UV light irradiation was sensitive to the DP of the PVBO block. More interestingly, the first derivatives of the time-dependent current clearly revealed Coulomb-blockade oscillation of the current flow, which also depended on the DP of the PVBO block. FFT analysis indicated that the current flowing through the PGNH as a function of time exhibited sawtooth oscillation. Current modulation of the PGNH by visible/UV light irradiation can be directly applied in amperometric UV sensors. In addition, the block-copolymer-mediated Coulomb-blockade transport in the graphene sheets provides a facile route to various Coulomb-blockade behavior–based applications, such as Coulomb-blockade-based transistors and thermometers.

## Methods

### Materials

Graphite flakes, potassium persulfate (≥99%), phosphorus pentoxide (≥98%), poly(ethylene glycol)methyl ether (average M_n_ ~5000), 3-VBA (97%), acetic anhydride (99%), sodium acetate (99%), and sulfuric acid (95.0–98.0%) were purchased from Sigma-Aldrich. Anisole (99%), PMDETA (99%), CuBr (99%), and hippuric acid (98%) were purchased from TCI. PDMS precursor and its curing agent (Sylgard 184) were purchased from Dow Corning.

### Synthesis of the block copolymer

PEO-*b*-PVBA: 3-VBA (2.287 mL, 18.0 mmol), PEO-Br (0.9 g, 0.18 mmol), PMDETA (37.58 µL, 0.18 mmol), and anisole (5.0 mL) were added to a Schlenk flask equipped with a magnetic stir bar. Oxygen was removed by three freeze–pump–thaw cycles, and then CuBr (25.82 mg, 0.18 mmol) was added under argon. Polymerization was conducted at 110 °C. Samples were removed by syringe at regular intervals (2, 4, and 6 h). The catalyst was removed by passing the solution through a neutral alumina column. The polymer samples were precipitated by adding the solution to cold diethyl ether, and the products were dried overnight under vacuum at room temperature. The DP of the PVBA block in the resulting PEO-*b*-PVBA was controlled at 25, 40, and 50 and was identified by ^1^H NMR spectroscopy. ^1^H NMR (300 MHz DMSO-d_6_, ppm): δ 9.95‒9.55 (1 H, s, CHO), δ 7.76‒6.52 (4 H, m, Ar‒H), 3.55‒3.45 (4 H, s, O‒CH_2_‒CH_2_), 3.37‒3.29 (6 H, s, ‒CH_3_‒C‒CH_3_), 1.95‒1.21 (3 H, s, aliphatic H). PEO_113_-*b*-PVBA_25_ (M_n_ = 29,380 g mol^‒1^, M_w_/M_n_ = 1.23), PEO_113_-*b*-PVBA_40_ (M_n_ = 42,870 g mol^‒1^, M_w_/M_n_ = 1.33), PEO_113_-*b*-PVBA_50_ (M_n_ = 61,400 g mol^‒1^, M_w_/M_n_ = 1.41).

PEO-*b*-PVBO: Typically, PEO_113_-*b*-PVBA_25_ (0.85 g, 2.2 mmol), hippuric acid (1.99 g, 11.1 mmol), sodium acetate (0.911 g, 11.1 mmol), and acetic anhydride (15.0 mL) were added to a round-bottom flask, heated at 120 °C for 4 h, and then stirred overnight at room temperature. Acetic anhydride was removed by vacuum evaporation, and the reaction mixture was precipitated in cold diethyl ether. The resulting polymer was dried overnight under vacuum at room temperature. ^1^H NMR (300 MHz DMSO-d_6_, ppm): δ 8.45‒5.82 (10 H, m, Ar‒H and vinyl‒H), 3.54‒3.46 (4 H, s, O‒CH_2_‒CH_2_), 3.40‒3.28 (6 H, s, ‒CH_3_‒C‒CH_3_), 2.10‒0.38 (3 H, s, aliphatic H). The isomerization of VBO unit induced by UV irradiation was confirmed by ^1^H NMR spectroscopy (Supplementary Fig. 7).

### Intercalation of PEO*-b-*PVBO into graphene

First, graphite was exfoliated using an acidic solution. Phosphorous pentoxide and potassium persulfate were dissolved in a sulfuric acid solution, and graphite flakes (the weight ratio of each reagent to graphite was 5:1) were mixed into the solution, which was then stirred for 40 min at 80 °C. The exfoliated graphite (EG) was thoroughly washed with distilled water and then heat-treated at 1000 °C for 90 min under nitrogen atmosphere (see Raman spectrum in Supplementary Fig. 8).

The EG was dispersed in distilled water at an EG-to-water weight ratio of 1:2000 by sonication. Subsequently, PEO*-b-*PVBO was added to the EG dispersion solution at a PEO*-b-*PVBO/EG weight ratio of 2:1. The PEO*-b-*PVBO/EG dispersion was sonicated to facilitate intercalation of the block copolymer into the graphene.

### Characterization

^1^H NMR spectra of the polymers in DMSO-d_6_ were collected on a Bruker Avance 300 MHz NMR spectrometer. The apparent molecular weights and molecular weight distributions were measured by GPC (Agilent Technologies, 1200 series) using a polystyrene standard with dimethylformamide as an eluent at 30 °C and a flow rate of 1.00 mL min^−1^.

SEM was used to observe the morphology of the block copolymer on graphene sheets using a ZEISS GeminiSEM 500 electron microscope; platinum sputter coating (10 mA, 10 s) was used to prevent charge build-up. UV–visible spectroscopy was used to analyze changes in the samples with increasing UV light irradiation time. An Optizen 2120UV spectrophotometer was used for the measurement. To measure the absorbance of the colloidal samples, all of the dispersed samples were diluted 20 times with distilled water.

### Electrical monitoring

A homemade measurement setup was built for the electrical monitoring under visible/UV irradiation (Supplementary Fig. 9). The current as a function of time at an applied voltage was measured using a Keithley 2636A sourcemeter. While the electrode ensemble was periodically exposed to UV and visible light, the current was measured at an applied voltage of 0.1 V. The wavelengths of the UV and visible light sources were 365 and 650 nm, respectively, at an irradiation power density of 4.2 mW cm^−2^.

## Supplementary information

Supplementary Information

## Data Availability

The data supporting this study are available in the paper and the Supplementary Information. If need, other relevant data may be available from the corresponding authors upon reasonable request.
