# Supplementary material for: Electrical monitoring of photoisomerization of block copolymers intercalated into graphene sheets
Source: Nat Commun. 2020 Mar 12;11:1324. doi: 10.1038/s41467-020-15132-z (PMC7067762; doi:10.1038/s41467-020-15132-z)
Supplement: Supplementary file 1 — Supplementary Information [file 41467_2020_15132_MOESM1_ESM.pdf]

*Supplementary information*

# Electrical monitoring of photoisomerization of block copolymers intercalated into graphene sheets

Semin Kim,[2] Thanh-Hai Le,[2] Yunseok Choi,[2] Haney Lee,[1] Eunseo Heo,[2] Unhan Lee,[2] Saerona Kim,[2] Subin Chae,[2] Yoong Ahm Kim[1,2] and Hyeonseok Yoon[1,2,*]

[1]Alan G. MacDiarmid Energy Research Institute & School of Polymer Science and Engineering, Chonnam National University, 77 Yongbong-ro, Gwangju 61186, South Korea

[2]Department of Polymer Engineering, Graduate School, Chonnam National University, 77 Yongbong-ro, Gwangju 61186, South Korea

Corresponding Authors:

*H. Yoon, hyoon@chonnam.ac.kr

**Photoluminescence decay behavior**

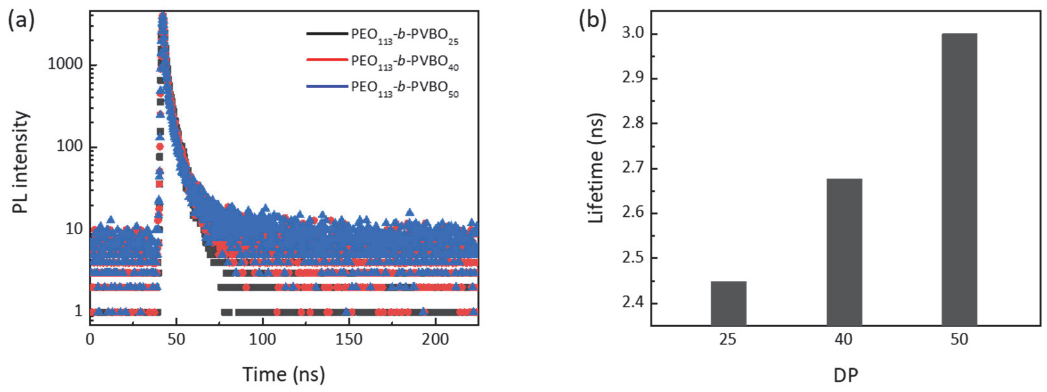


**Supplementary Figure 1: Photoluminescence decay behavior of the block copolymers.** (a) Time-resolved PL decay curves recorded in water and (b) histogram showing the calculated lifetimes.

**UV-visible absorption spectra of PVBO homopolymer**

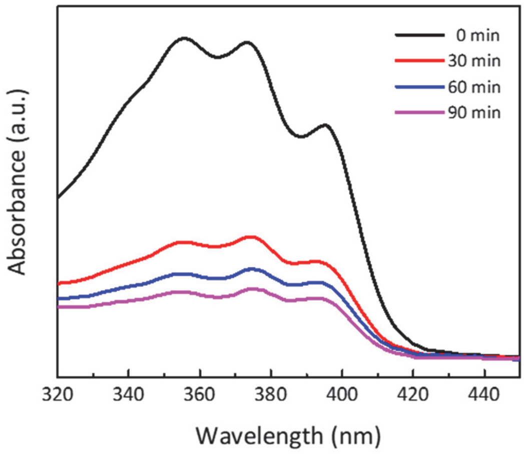


**Supplementary Figure 2: UV-visible absorption spectra of aqueous dispersions of PVBO only under UV irradiation for 90 min (recorded at 0, 30, 60, and 90 min).**

**UV-visible absorption spectrum of graphene_only**

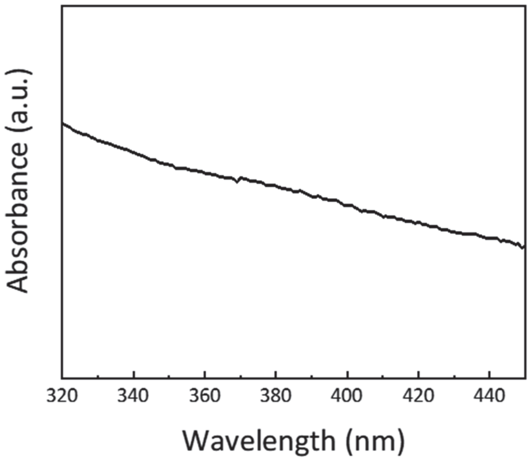


**Supplementary Figure 3: Absorbance spectrum of the graphene colloid dispersed in water.** The graphene dispersion showed no notable absorbance peak, even after UV irradiation.

**Correlation between the UV/visible absorbance and the photoisomerization kinetics**

*Z–E* photoisomerization can be described as follows:

$$(Rate) = -\frac{\Delta[Z]}{\Delta t} = k[Z] \quad \text{(S1)}$$

Integration of the above kinetic equation results in

$$\ln[Z(t)] = -kt + \ln[Z]_0 \quad \text{(S2)}$$

where $[Z(t)]$ is the concentration of the $Z$ isomer at time $t$, $[Z]_0$ is the initial concentration of the $Z$ isomer, and $k$ is the rate constant of the reaction. Eq. S2 can be rearranged as

$$\frac{[Z(t)]}{[Z]_0} = e^{-kt} \quad \text{(S3)}$$

$$A(t) = A_{Z,t} + A_{PEO,t} + A_{Graphene,t} \quad \text{(S4)}$$

$$A_0 = A_{Z,0} + A_{PEO,0} + A_{Graphene,0} \quad \text{(S5)}$$

where the measured absorbance $A(t)$ is the sum of the components $A_{Z,t}$, $A_{PEO,t}$, and $A_{Graphene,t}$, which are the absorbance values of the $Z$ isomer, PEO, and graphene at time $t$, respectively. By dividing $A(t)$ by the initial absorbance $A_0$, the following equation is obtained:

$$\frac{A(t)}{A_0} = \frac{(A_{Z,t}+A_{PEO,t}+A_{Graphene,t})}{(A_{Z,0}+A_{PEO,0}+A_{Graphene,0})} \quad \text{(S6)}$$

Letting

$$p = \frac{A_{PEO,t}}{A_{Z,0}} + \frac{A_{Graphene,t}}{A_{Z,0}} \; [\because A_{PEO,t}, A_{Graphene,t} = (constant)]$$

$$q = (1 + \frac{A_{PEO,0}}{A_{Z,0}} + \frac{A_{Graphene,0}}{A_{Z,0}})$$

and substituting $p$ and $q$ into Eq. S6 yields

$$\frac{A(t)}{A_0} = \frac{(A_{Z,t}/A_{Z,0}+p)}{q} \quad \text{(S7)}$$

According to the Beer–Lambert Law, Eq. S7 can be written as

$$\frac{A(t)}{A_0} = \left(\frac{1}{q}\right)\left(\frac{\varepsilon_Z l[Z(t)]}{\varepsilon_Z l[Z]_0}\right) + \left(\frac{p}{q}\right) = \left(\frac{1}{q}\right)\left(\frac{[Z(t)]}{[Z]_0}\right) + \left(\frac{p}{q}\right) \quad \text{(S8)}$$

where $\varepsilon_Z$ is the molar absorptivity of the $Z$ isomer, and $l$ is the path length of the sample. Both components could be reducible.

Rearranging and substituting Eq. S3 yields the following equation.

$$\frac{[Z(t)]}{[Z]_0} = \left(q\frac{A(t)}{A_0} - p\right) = e^{-kt} \quad \text{(S9)}$$

This is rearranged as

$$\frac{A(t)}{A_0} = \frac{p}{q} + \left(\frac{1}{q}\right)e^{-kt} \quad \text{(S10)}$$

and then

$$\alpha = \frac{A_{Z,0}}{A_{Z,0}+A_{PEO,0}+A_{Graphene,0}} = \frac{1}{q}$$

$$\beta = \frac{A_{PEO,t}+A_{Graphene,t}}{A_{Z,0}+A_{PEO,0}+A_{Graphene,0}} = \frac{p}{q}$$

are substituted into Eq. S10 to obtain

$$\frac{A(t)}{A_0} = \beta + \alpha e^{-kt} \quad \text{(S11)}$$

**Time-dependent density functional theory calculation**

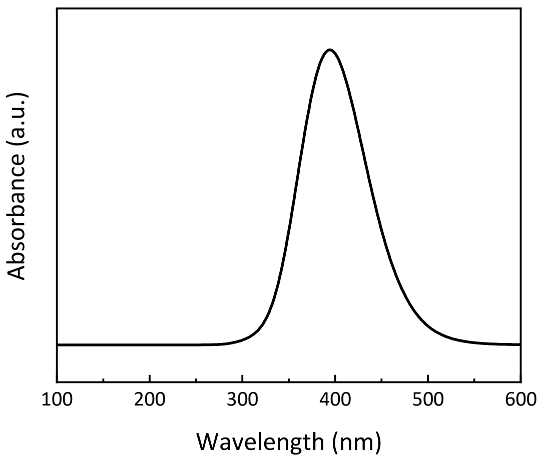


**Supplementary Figure 4: UV-visible spectrum of the BO moiety obtained through time-dependent DFT calculation (B3LYP, 6-311+g(d,p), water medium, single states).** A broad absorption occurred around 400 nm (max. 3.15 eV (394 nm)).

**Electrical response of graphene alone**

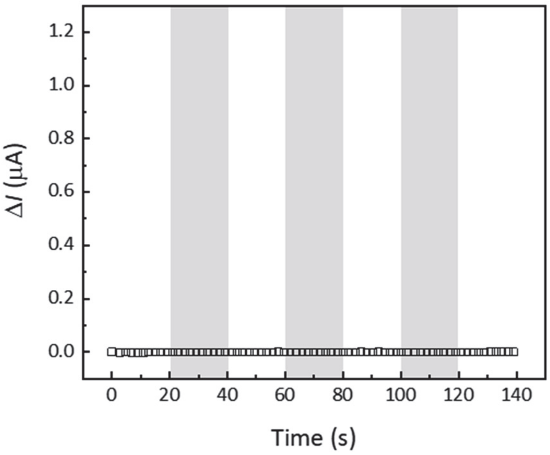


**Supplementary Figure 5: Electrical response of EG recorded at an applied voltage of 0.1 V as a function of time upon periodic exposure to visible (white region) and UV (gray region) light.** EG was insensitive to both types of light.

**Frequency domain: two different oscillations**

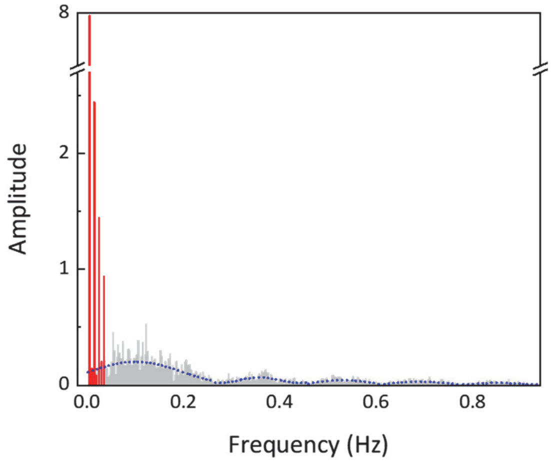


**Supplementary Figure 6: Example of frequency peak $f_1$ (red), frequency band $f_2$ (blue), and their harmonics.**

**NMR spectra of the block copolymer isomers**

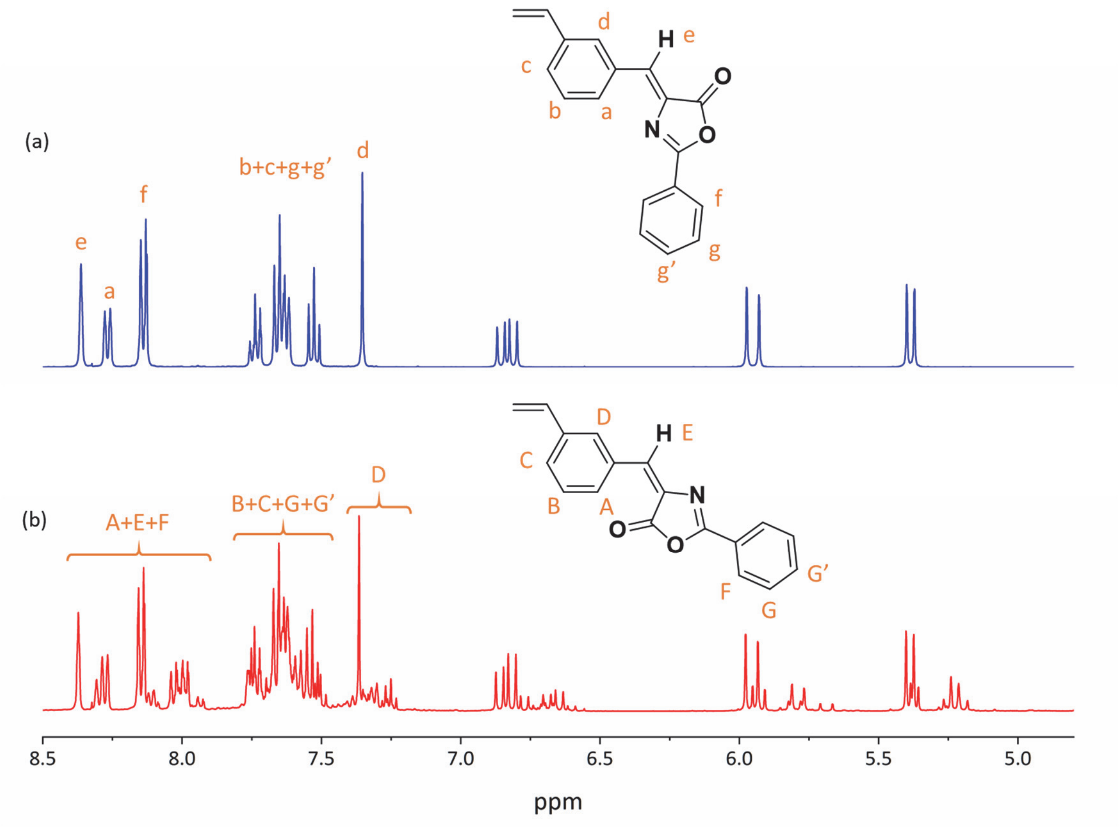


**Supplementary Figure 7: Characterization of the isomers by NMR spectroscopy.** $^{1}$H NMR spectra of VBO measured (a) before and (b) after UV irradiation.

**Characteristics of graphene precursor**

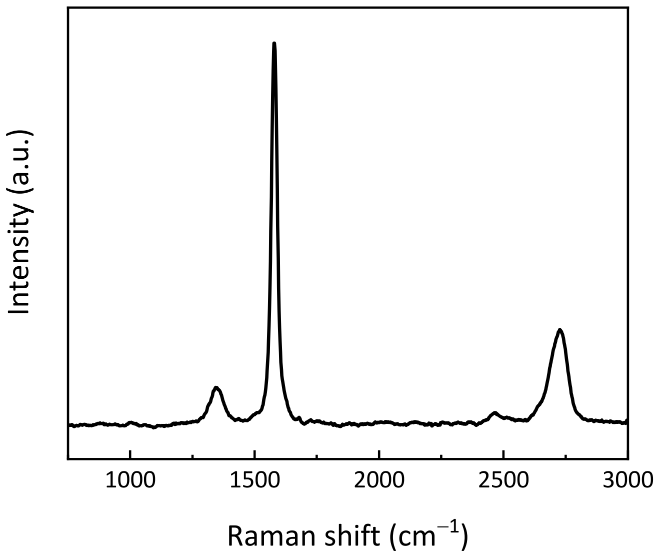


**Supplementary Figure 8.** Raman spectrum of the graphene precursor recorded with 532.13 nm excitation using a JASCO NRS-5100 spectrophotometer. The characteristic *D*, *G*, and *G*′ peaks were found at ~1340, ~1580, and ~2730 $cm^{-1}$. The intensity ratio of *D* peak-to-*G* peak was 0.07 and the four probe conductivity of the graphene precursor pellet was measured to be 550 S $cm^{-1}$.

**Experimental setup for the measurement**

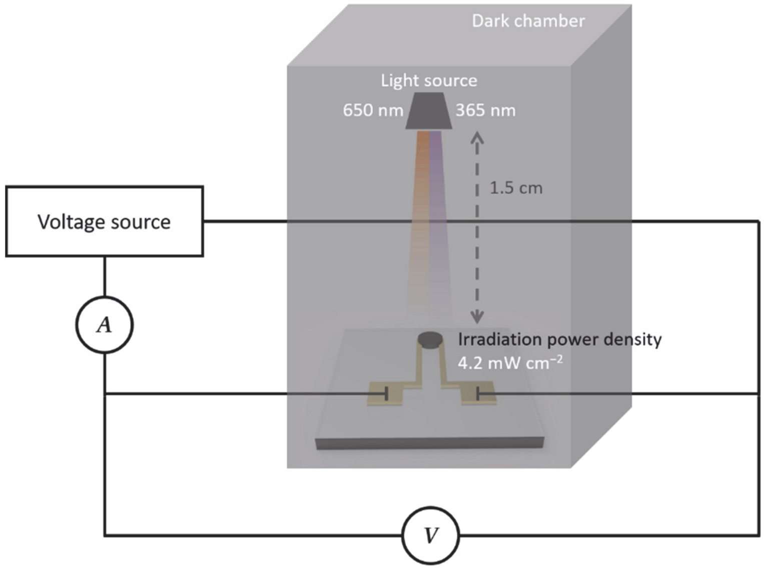


**Supplementary Figure 9.** Experimental setup for the electrical monitoring of the PGNH electrode. The width of the gold finger in the used electrode was 500 μm and the inter-finger gap was 1.5 mm.
